# Coffee berry borer (*Hypothenemus hampei*) (Coleoptera: Curculionidae) development across an elevational gradient on Hawai‘i Island: Applying laboratory degree-day predictions to natural field populations

**DOI:** 10.1371/journal.pone.0218321

**Published:** 2019-07-17

**Authors:** Lindsey J. Hamilton, Robert G. Hollingsworth, Mehana Sabado-Halpern, Nicholas C. Manoukis, Peter A. Follett, Melissa A. Johnson

**Affiliations:** 1 United States Department of Agriculture—Agricultural Research Service, Daniel K. Inouye US Pacific Basin Agricultural Research Center, Hilo, Hawaii, United States of America; 2 Oak Ridge Institute for Science and Education, Oak Ridge, Tennessee, United States of America; Fred Hutchinson Cancer Research Center, UNITED STATES

## Abstract

Coffee berry borer (CBB, *Hypothenemus hampei*) (Coleoptera: Curculionidae: Scolytinae) is the most destructive pest of coffee worldwide. Information on CBB development times can be used to predict the initiation of new infestation cycles early in the coffee-growing season and thus inform the timing of insecticide applications. While laboratory estimates of CBB development under constant conditions exist, they have not been applied under the heterogeneous environmental conditions that characterize many coffee-growing regions. We measured CBB development times and abundance in commercial coffee farms across an elevational gradient on Hawai‘i Island and applied thermal accumulation models from previous laboratory studies to test their fit to field data. Artificial lures were used to infest coffee berries at five farms ranging in elevation from 279–792 m, and weather variables were monitored at macro (farm-level) and micro (branch-level) scales. CBB development was followed in the field from the time of initial berry infestation by the founding female through the development of F1 mature adults. Mean development time from egg to adult across all sites was 38.5 ± 3.46 days, while the mean time required for the completion of a full life cycle (from time of infestation to presence of mature F1 females) was 50.9 ± 3.35 days. Development time increased with increasing elevation and decreasing temperature. Using macro-scale temperature data and two different estimates for the lower temperature threshold (14.9°C and 13.9°C), we estimated a mean requirement of 332 ± 14 degree-days and 386 ± 16 degree-days, respectively, from the time of berry infestation to the initiation of a new reproductive cycle in mature coffee berries. Similar estimates were obtained using micro-scale temperature data, indicating that macro-scale temperature monitoring is sufficient for life-cycle prediction. We also present a model relating elevation to number of CBB generations per month. Our findings suggest that CBB development times from laboratory studies are generally applicable to field conditions on Hawai‘i Island and can be used as a decision support tool to improve IPM strategies for this worldwide pest of coffee.

## Introduction

Coffee berry borer (“CBB”, *Hypothenemus hampei* Ferrari) (Coleoptera: Curculionidae: Scolytinae) is the most destructive pest of coffee worldwide, causing enormous economic losses through direct damage to coffee beans [1, 2, 3]. Endemic to Africa, this highly invasive beetle is now found in all coffee-producing countries (the single exception is Nepal) and reduces both the yield and quality of coffee products [4, 5]. Adult females bore into the central disc of the developing green berries and into the coffee bean itself, where they excavate galleries in which to lay their eggs [6, 7, 8]. Females can lay >100 eggs in a single bean during a three-week period [8]. Inside the bean, the CBB develops through four major life stages: egg, larva (first and second instars), pupa, and adult (teneral and mature). CBB development occurs over 1–2 months depending on temperature and berry moisture [6, 7, 8]. Male offspring inseminate female siblings, and the mated females leave and search for a new berry in which to lay eggs or remain in the natal berry and begin reproduction [6, 7, 8]. The founding female remains with her progeny and does not leave the berry unless disturbed [6].

Sustainable strategies for managing this pest are becoming increasingly important because highly toxic insecticides that have been widely used to control CBB, such as endosulfan and chlorpyrifos, have been banned or are close to being phased out in most coffee-growing regions amid concerns for human and environmental health [9, 10], as well as evidence for insecticide resistance [11, 12]. Integrated Pest Management (IPM) programs to control CBB are therefore urgently needed in many countries. Effective IPM programs will likely include a combination of control measures such as sanitation, pruning, biocontrol, and biopesticides [13, 14, 15]. While extensive work has been done to elucidate the most effective way of implementing each of these measures in countries such as Colombia (which has a long history of robust CBB management), research focused on improving the effectiveness of control measures is still needed in regions for which CBB is a recent arrival. Studies aimed at improving control measures are particularly important for regions that experience high labor and production costs, such as Hawaii and Puerto Rico. For example, biopesticides like the entomopathogenic fungus *Beauveria bassiana* are commerically available to control CBB in Hawaii [15] and Puerto Rico [16], but the frequency, dosage, and timing of sprays is usually arbitrary [17]. To optimize results and improve the cost-effectiveness of these biopesticides, sprays must be accurately timed with the emergence of adult CBB females seeking new berries to infest, because this is when they are exposed and most vulnerable [17].

Information on the development rate of CBB under natural field conditions can be used to infer emergence of females and thus inform optimal spray times. Insects, as poikilotherms, have their developmental rates primarily determined by temperature, so population dynamics and the timing of infestation cycles in agriculturally important crops are often temperature-driven [18]. Despite its high economic impact as a crop pest [2, 19], few studies have examined the relationship between CBB development rate and temperature in the field. Field experiments on CBB in Mexico that used artificial infestation reported a 49-day development time from infestation to the appearance of F1 adults at a mean daily temperature of 20.9˚C, and a 42-day development time at 23˚C [20]. Ruiz-Cárdenas and Baker [21] also used artificial infestation in Columbia to estimate CBB development time from first egg to the first mature F1 female, and reported a development time of 49 days in field sites with mean daily temperatures ranging from 20.7–21.6˚C.

Laboratory studies that report thermal unit accumulation thresholds (“degree days”) for CBB [22, 23, 24] provide data that is an essential starting point for improving site-specific timing of control practices. Degree-day models relate insect development (time in stage) to ambient temperature such that higher temperatures result in decreased time in stage (increased development rate, defined as the reciprocal of time in stage), up to a certain temperature threshold where development halts [25]. A temperature one degree above the lower developmental threshold for 24h would result in the accumulation of one degree-day. From laboratory studies conducted under constant temperatures, the threshold number of degree-days needed for a stage transition or complete life cycle in CBB has been determined [22, 23, 24]. However, the reliability of these degree-day predictions under variable field conditions has not been tested.

In Hawaii, coffee is a relatively small but profitable industry. With its unique geographic origin and high-quality beans, Hawaii coffee commands premium prices on the world specialty market [26, 27]. CBB was first detected in 2010 on Hawai‘i Island [28], where there are approximately 800 coffee farms [29]. Since its introduction CBB has continued to spread across the state and was recently detected on the neighboring Hawaiian Islands of O‘ahu (2014) and Maui (2016). One of the major difficulties in managing this pest in Hawaii is that the land area suitable for coffee agriculture is extremely variable over short distances. Coffee is grown in Hawaii on flat coastal plains and rugged mountain slopes, the altitude spans from sea level to 800 m, the volcanic soils vary in age and nutrient composition, and rainfall can range from ~760–2540 mm a year [30]. The extreme variability in climate and topography across Hawaii’s coffee-growing regions necessitates a landscape-level approach to improving IPM.

In the present study, we collected weather data at both macro- (farm-level) and micro-scales (branch-level) from commercial coffee farms spanning an elevational gradient of 600 m on Hawai‘i Island, and employed a novel technique using alcohol lures to initiate infestation of coffee berries in the field by naturally-occuring adult females. We followed CBB development in the field from the time of infestation by founding females through to the development of mature F1 adults. Our objective was to compare development rates observed in the field with those predicted from previous studies under constant conditions, in order to assess the fit of degree-day models based on those laboratory data to the heterogeneous environmental conditions that characterize Hawaii’s coffee-growing regions. Findings from this study can be applied to coffee-growing countries around the world to improve the timing and efficacy of pesticide sprays as part of a multi-faceted IPM approach for this invasive beetle.

## Materials and methods

### Study sites

**Fig 1:**
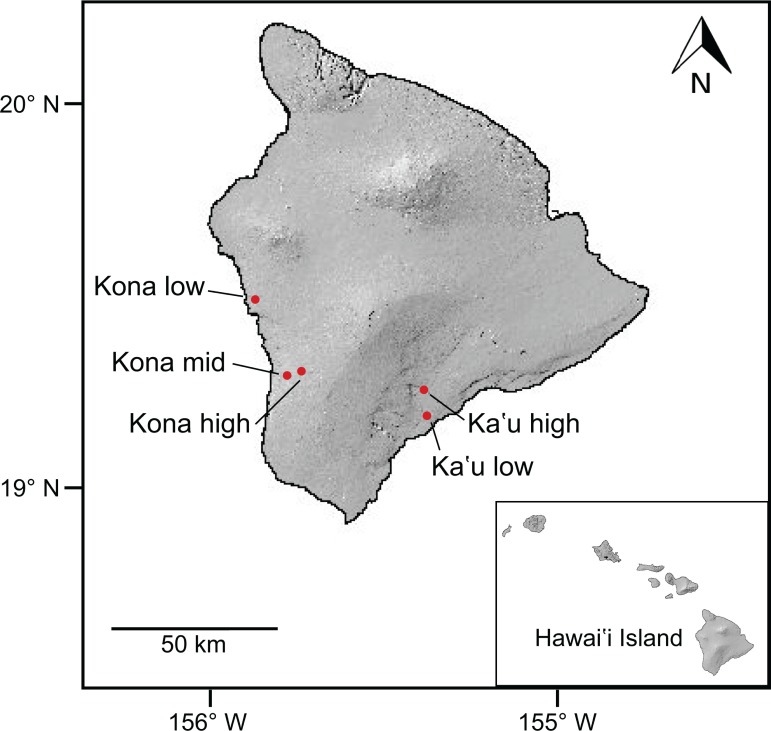
Map of Hawai‘i Island showing locations of the five study sites. Three sites were located in the Kona district on the west side of the island, and two sites were located in the Ka‘u district on the southeast side of the island. Kona sites represented low (427 m), mid (610 m), and high (792 m) elevations, while Ka‘u sites represented low (279 m) and high (778 m) elevations. Inset map shows the main Hawaiian Islands and the location of Hawai‘i Island within the archipelago.

Five commercial coffee farms were selected across an elevational gradient within two coffee-growing regions, Kona and Ka‘u, on Hawai‘i Island (Fig 1). Kona is located on the west side of Hawai‘i Island, and currently has ~1600 ha of coffee in production (S. Shriner, pers. comm.). The climate of Kona is characterized by hot sunny mornings and cool, cloudy afternoons with an average annual temperature of 24°C [30]. Winter temperatures and rainfall in Kona are low relative to the rest of Hawai‘i Island, resulting in a period of dormancy in the coffee. Flowering typically occurs from late February–April, following heavy rainfall events. Ka‘u is located on the southeast side of Hawai‘i Island and has ~200 ha of coffee in production (S. Shriner, pers. comm.). Ka‘u experiences relatively constant temperatures (average annual temperature of 22°C) and rainfall, resulting in year-round flowering and fruiting [30]. In Kona, we selected sites at three elevations: low (427 m), mid (610 m), and high (792 m); sites selected in Ka‘u represented low (279 m) and high (778 m) elevations (Fig 1). Four of the five sites were planted with *Coffea arabica* var. *typica* that was grown in full sun. The Ka’u low site was planted with *Coffea arabica* var. *catuai* and had sparsely planted *Samanea saman* (monkeypod) trees that provided shade to a small fraction of the coffee plants. Ground cover varied among sites from soil to rocks, grass, or perennial peanut.

### Lure infestations

To estimate the rate of CBB development in the field, we initiated coffee berry infestation using alcohol lures in September 2017 as described below. Permission was first obtained from each private land owner to conduct the study. We then scouted each site to identify areas within the farms that had high CBB infestation. In these areas, we selected 23–30 branches meeting the following requirements: 1) the majority of berries had reached the mature green stage, 2) there were few to no infested berries present, 3) there were ~100 berries, and 4) they were in close proximity to other branches that had high numbers of infested berries. Branches selected for lure infestation were located at various heights throughout the low, mid, and top portions of the trees. Prior to initiating infestations, we removed any infested berries (berries with an entrance hole in the central disc) from the selected branches. We prepared an attractant consisting of 3:1 methanol/ethanol in the laboratory and placed 10 mL of the attractant into semi-permeable plastic bags (2 mil, 3x4 inch) to use as lures. We attached a paperclip to each lure bag and used the clips to hang lures every three nodes on each branch to ensure that the attractant was in close proximity to all berries.

One week later, we checked branches for new infestations and each newly infested berry was marked with a gel pen. Of the 23–30 branches fitted with lures, we selected 10 branches to track CBB development based on sufficient numbers of new infestations (≥ 32 infested berries/branch; Table 1). Given that there were insufficient numbers of infestations at the three Kona sites after the first week, we opted to leave lures on branches for a second week at these sites, then we counted the number of newly infested berries and marked them at the end of week two with a different color. At the high and mid-elevation Kona sites, we pooled infestations from both weeks to achieve the desired number of infestations, while at the low-elevation Kona site infestations from the second week were sufficient, and first-week infestations were not utilized.

**Table 1:** Summary of infestation results using 3:1 methanol/ethanol lures hung from 10 selected branches per study site. Lures were left on branches for 1–2 weeks in order to attain sufficient numbers of infested berries per branch (≥ 32).

Study site	Elevation (m)	Lure exposure time	Berries per branch (mean ± se)	Infested berries per branch (mean ± se)	Infestation (%)
Kona High	792	2 weeks	124.3 ± 7.0	47.5 ± 5.9	40.3 ± 6.1
Kona Mid	610	2 weeks	119.8 ± 9.6	33.9 ± 5.8	29.3 ± 5.2
Kona Low^a^	427	1 week	130.5 ± 14.9	47.1 ± 6.7	39.8 ± 6.8
Ka‘u High	778	1 week	92.1 ± 6.5	72.5 ± 2.7	80.7 ± 3.9
Ka‘u Low	279	1 week	118.3 ± 12.3	106.3 ± 8.0	91.7 ± 2.5

^a^First round of infestations not represented in this table.

### Micro- and macro-scale weather

At each study site we monitored weather variables in two ways, one to capture micro-scale weather on branches and another to measure macro-scale weather on farms. To monitor micro-scale weather, 10 branches per farm were each equipped with a light intensity meter (“solar pendant”, UA-002-64, Onset Computer Corporation, Bourne, USA) and temperature/relative humidity logger (Thermocrons iButton type DS1923; Maxim/Dallas Semiconductor Corp., USA), set to record measurements hourly. Weather stations were also installed at each site to monitor farm (macro-scale) weather. At the high and mid-elevation sites in Kona, weather stations included a temperature/relative humidity logger (U23 Pro v2, Onset Computer Corporation, Bourne, MA, USA) enclosed within a solar radiation shield (RS3, Onset Computer Corporation, Bourne, MA, USA), and a solar pendant. The two Ka‘u sites had HOBO 3G cellular remote monitoring stations (RX3000, Onset Computer Corporation, Bourne, USA) equipped with a temperature/relative humidity sensor (S-THB-M002, Onset Computer Corporation, Bourne, MA, USA), and solar radiation sensor (silicon pyranometer S-LIB-M003, Onset Computer Corporation, Bourne, MA, USA) enclosed within a solar radiation shield (RS3, Onset Computer Corporation, Bourne, MA, USA). The low-elevation Kona site was monitored using a RV50 cellular modem equipped logger (Campbell Scientific, Inc., Logan, UT, USA) with a temperature/relative humidity sensor (HC2S3, Rotronic Instrument Corporation, Hauppauge, NY, USA) and a solar radiation sensor (200LX, LI-COR, Lincoln, NE, USA) enclosed within a solar radiation shield (RS3, Onset Computer Corporation, Bourne, MA, USA). Each weather station recorded data hourly, which was then averaged for each day to estimate maximum, mean, and minimum daily values for each variable in order to characterize each site.

### Rates of development and number of individuals per fruit

From each of the 10 branches, we collected 2–6 infested berries weekly, for a total of 40 infested berries collected per site per week over 8–10 weeks. For the mid and high-elevation Kona sites we collected berries from the first and second rounds of infestation, ensuring that similar numbers of berries were collected from each round (i.e., ~20 berries from each of the two rounds). After collection, we stored berries at 14°C to suspend beetle development. Within 24 hrs of collection, berries were weighed and dissected using a stereomicroscope following the protocol described in Johnson et al. [31]. For each berry, the total number of each life stage (eggs, larvae, pupae, teneral adults, and mature adults) was recorded. Adults are termed ‘teneral’ when they first emerge from the pupa stage and their exoskeletons have not yet hardened, causing them to be pale in color. We used the R package ‘stats’ v. 3.5.0 [32, 33] to assess data normality with a Shapiro-Wilk test, and then conducted a Spearman’s rank correlation test for non-normal data to estimate the relationship between temperature and the total number of CBB in all life stages per berry.

### Berry moisture content

Following dissections, berries were placed in labeled containers and dried in an oven at 27°C for 12 hours or until they reached a constant weight. Dry berry weight (g) was recorded, and berry moisture (%) was calculated by dividing the dry weight by the fresh weight. This was done to ensure that berry development was sufficient for gallery construction and oviposition [34].

### Degree-day calculations

Cumulative degree-days were calculated by site using micro- or macro-scale temperature data and the lower temperature threshold (LTT) for CBB development determined under constant conditions in two separate studies. To calculate degree-day accumulation, the LTT of 13.9°C [24] and 14.9°C [22] were subtracted from each hourly temperature reading and the resulting values were summed, divided by 24 hrs, and an average calculated for each day. For micro-scale data, hourly temperatures from all 10 branches per site were averaged for degree-day calculations. Given that berry infestation resulting from lures was only checked on a weekly basis, we estimated the time of infestation to be the mid-point in the week (i.e., 3.5 days post-lure setup). We calculated micro- and macro-scale values for the total cumulative degree-days elapsed for one CBB development cycle by summing daily degree-day accumulation from the estimated time of infestation to the first appearance of mature F1 adults. Daily degree-day accumulations were also used to predict the number of days required for CBB to develop from an egg to an adult under field conditions using the pre-reproductive thermal requirements of 262.47 and 299 degree-days established by Jaramillo et al. [22] and Giraldo-Jaramillo et al. [24], respectively. We assessed the data for normality using a Shapiro-Wilk test in the R ‘stats’ package [32, 33]. We then compared predicted development times with those from field observations for each site, using branches as replicates and a Mann-Whitney U test for non-normal data in the R ‘stats’ package [32, 33].

To estimate the number of CBB generations per season along an elevational gradient, accumulated degree-days were calculated using macro-scale weather data from January–December 2017 for 12 commercial coffee farms on Hawai‘i Island (Kona = 8, Ka‘u = 4) ranging in elevation from 204–778 m. These farms are part of a larger CBB monitoring program, and are equipped with weather stations that record temperature, solar radiation, relative humidity, and rainfall. Due to seasonal differences between Kona and Ka‘u coffee production, rates were calculated as the average number of generations per month by dividing total annual accumulated degree-days by 30.42 days. We assessed the data for normality using a Shapiro-Wilk test and tested for equal variances with an F-test in the R ‘stats’ package [32, 33]. We calculated the number of generations per season using two different estimates for the LTT (13.9°C and 149°C) and compared these using a student’s t-test for normal data in the R ‘stats’ package [32, 33].

## Results

### Lure infestations

Of the 50 branches selected for lure infestations across five study sites, 38% were located on the lower third of the trees, 58% in the middle third, and 4% on the upper third. Infestations one week after lure deployment were substantially higher at the two Ka‘u sites relative to all three Kona sites (Table 1). Higher infestation on branches at the Ka‘u sites was likely due to higher overall infestation on these farms (Johnson et al., unpubl. data).

### Micro- and macro-scale weather

Branch (micro) and farm (macro) weather data are summarized in Table 2. In general, maximum (*Tmax*) and mean (*Tmean*) daily temperatures were higher and minimum daily temperatures (*Tmin*) were lower on branches relative to values recorded at the farm level. Differences in micro and macro *Tmean* and *Tmin* were < 1°C for all sites. In contrast, differences in micro and macro *Tmax* ranged from 1.30–4.14°C across sites. Branches also experienced greater maximum relative humidity (*RHmax*) and lower mean (*RHmean*) and minimum (*RHmin*) relative humidity compared to farms. Differences in RH among sites were generally < 2%; however, differences in *RHmin* ranged from 1.43–13.42% across sites. Substantially lower light intensities were recorded for branches relative to farms; across all sites the mean daily solar intensity on branches was 57% lower than that recorded for farms (Table 2).

**Table 2:** Mean daily values (maximum, mean, and minimum) for macro- and micro-scale weather variables by site from September–November 2017. Micro-scale data represents the mean from 10 sample branches at each site.

Study site	Macro/ Micro	*Tmax* (°C)	*Tmean* (°C)	*Tmin* (°C)	*RHmax* (%)	*RHmean* (%)	*RHmin* (%)	Solar Radiation (Lux)	Rainfall (mm)
Kona High	Macro	24.75	20.30	16.78	99.98	95.44	81.33	22106.95	5.72
	Micro	26.82	20.67	16.86	100.00	95.02	76.71	9481.71	
Kona Mid	Macro	25.27	21.31	17.85	99.21	92.52	78.98	22908.86	4.94
	Micro	29.41	22.09	17.86	99.56	89.87	65.56	19202.96	
Kona Low	Macro	26.54	22.76	19.70	95.41	82.32	66.68	21851.52	7.16
	Micro	29.82	23.07	18.98	98.05	86.45	65.25	11494.51	
Ka‘u High	Macro	26.05	19.93	16.05	98.52	88.96	68.12	18131.62	9.26
	Micro	29.45	20.17	15.31	99.45	88.36	58.56	13035.42	
Ka‘u Low	Macro	28.67	23.25	18.59	95.35	82.87	63.60	22174.89	4.29
	Micro	29.97	23.14	18.47	93.31	81.62	60.47	7390.65	

### Rates of development and number of individuals per berry

**Fig 2:**
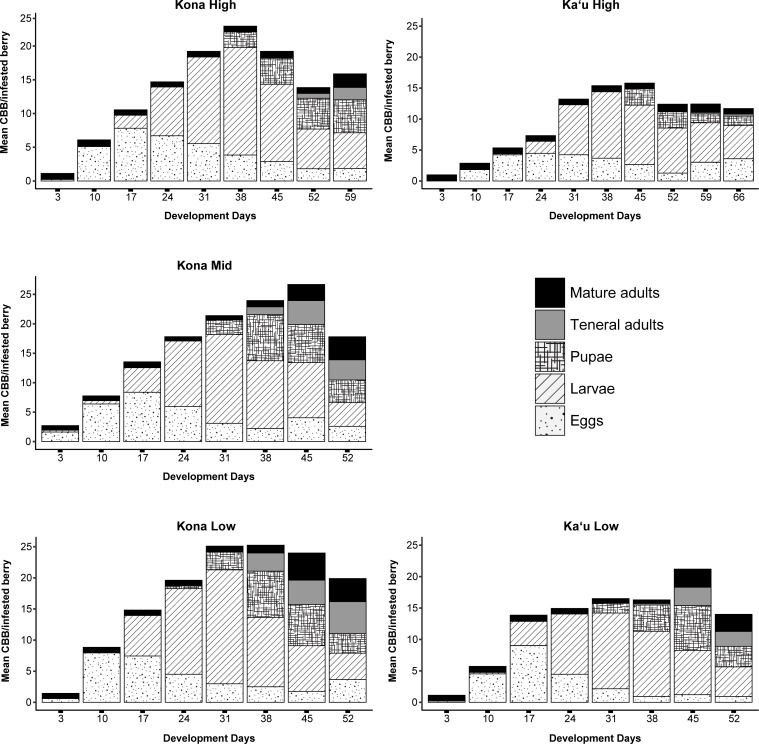
Mean numbers of each CBB life stage documented from dissections of infested coffee berries. Estimated infestation dates ranged from September 8–15 2017, and berry collections occurred on a weekly basis until November 1–15, 2017.

At all five sites, eggs were found in berries on the first collection date (Fig 2), meaning founding females had been lured to the berries, bored into the endosperm, and begun reproduction. We estimated a mean of 7.6 days from the estimated time of infestation to the time of first eggs across all branches at the five study sites. Mean development time from egg to teneral adult across all sites was 38.5 ± 3.46 days (mean ± 1 SE), while the mean time required for the completion of a full life cycle (estimated time of infestation to presence of mature F1 offspring) was 50.9 ± 3.35 days, with longer development times at higher elevations (Table 3).

**Table 3:** Summary of mean daily temperatures at the micro (branch) and macro (farm) scales and the 95% Confidence Interval (CI), as well as days to first appearance of each life stage averaged across all 10 branches at each study site.

Study site	Micro temp (95% CI)	Macro temp (95% CI)	Egg	Larvae	Pupae	Teneral adult	Mature adult
Kona High	20.67 (16.12–26.80)	20.30 (15.92–24.75)	8.6	8.4	32.2	45.50	47.60
Kona Mid	22.09 (17.34–28.90)	21.31 (17.18–25.36)	5.9	7.6	25.7	35.00	43.40
Kona Low	23.07 (18.7–29.29)	22.76 (19.30–26.70)	6.5	9.1	23.1	31.50	35.70
Ka‘u High	20.17 (14.84–29.74)	19.93 (15.47–25.90)	9.3	14.0	35.7	49.00	51.33
Ka‘u Low	23.14 (17.73–30.15)	23.25 (18.10–29.00)	7.9	7.0	25.2	32.67	38.11

^a^ Measured from the day that is midway between date of lure installation and first collection.

Across all sites the number of individuals in each life stage per berry was as follows: eggs = 7.52 ± 0.64, larvae = 14.42 ± 1.11, pupae = 5.98 ± 0.79, teneral adults = 2.84 ± 0.69, mature adults = 2.90 ± 0.45. The total number of individuals (the sum of all life stages) per berry was generally lower at high-elevation sites compared to low-elevation sites throughout the sampling period (Fig 2); however, we did not find a relationship between temperature and mean number of individuals per berry (r = 0.4, p = 0.52).

### Berry moisture content

Mean berry moisture across all five sites was 69.38% ± 0.24%. The vast majority of infested coffee berries that we sampled had a dry matter content of ~30%, which is well above the minimum dry matter content of 20% required for gallery construction and reproduction [34].

### Degree-day calculations

**Fig 3:**
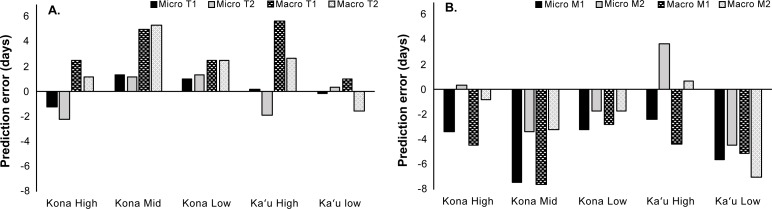
Difference between observed development times under field conditions (micro-temperature = branches and macro-temperature = farms) at five coffee farms on Hawai‘i Island and predicted development times (in days) under constant laboratory conditions. (A). Comparison of teneral adult development times between Jaramillo et al. [23; T1] and Giraldo-Jaramillo et al. [24; T2], (B) Comparison of mature adult development times between Jaramillo et al. [23; M1] and Giraldo-Jaramillo et al. [24; M2]. Significant differences between observed and predicted development times are indicated as follows: * = p < 0.05, ** = p < 0.01, *** = p < 0.001.

Across all five sites, we estimated 332 ± 16 degree-days (LTT = 14.9 ˚C) and 386 ± 14 degree-days (LTT = 13.9˚C) from the time of berry infestation to the appearance of the first mature F1 adult. A significant difference was found between observed and predicted development rates from egg to F1 teneral adults at the Kona mid-elevation site using macro-scale temperatures (W = 0, p = 0.001 and 0.002; Fig 3A). The observed development rate from egg to mature F1 adult in the field was also significantly different from the predicted development rate using degree-day models for the Kona mid-elevation site (micro: W = 30, p = 0.006; macro: W = 30, p = 0.003 and 0.005) and the Ka‘u low-elevation site (micro and macro: W = 72, p = 0.005; Fig 3B).

**Fig 4:**
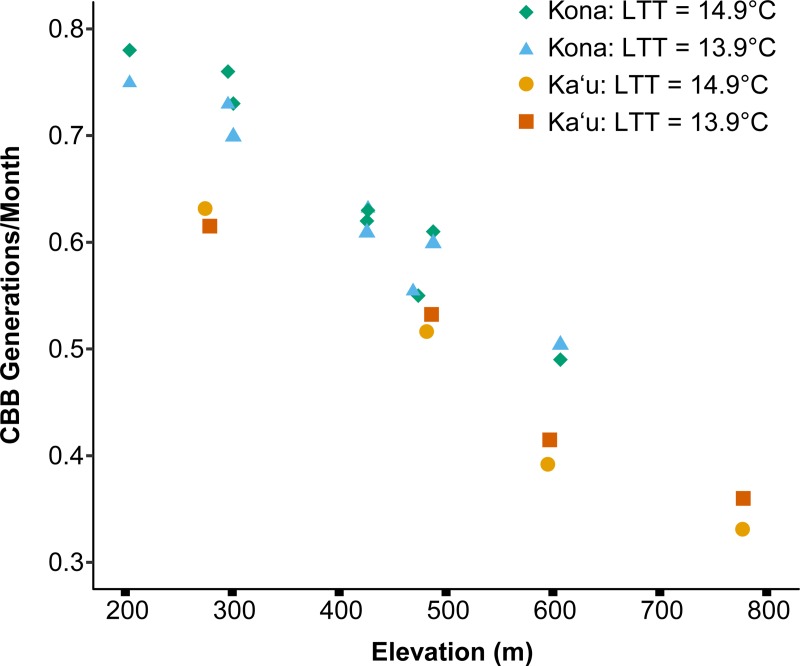
Mean number of CBB generations per month assuming a 332-degree day development requirement (LTT = 14.9°C) and a 386-degree day development requirement (LTT = 13.9°C) from berry infestation by the founding female to maturation of F1 offspring. Calculations were based on temperature data collected from January–December 2017 at 12 commercial coffee farms ranging in elevation from 204 to 778 m on Hawai‘i Island (Kona = 8, Ka‘u = 4).

Across 12 farms we estimated a mean of 0.59 ± 0.04 CBB generations per month using a LTT of 14.9°C, and a mean of 0.58 ± 0.03 CBB generations per month using a LTT of 13.9°C (t = 0.17, df = 21.51, p = 0.87). A significant negative correlation (tau = -0.81, p < 0.001) was found between elevation and the estimated number of generations per month (Fig 4). The estimated number of generations per month was observed to be slightly higher for sites in Kona relative to those in Ka‘u at similar elevations (Fig 4). Combining both areas, we found a strong relationship between elevation and CBB generations per month with an R^2^ = 0.89, predicted by the linear equation y = -0.0008x + 0.95, where y = CBB generations per month and x = elevation in meters (F_1, 10_ = 77.13, p < 0.001).

## Discussion

### Micro- and macro-scale weather

In general, our micro-scale sensors recorded more extreme temperature and humidity values across the five study sites. The higher maximum temperatures recorded by the micro-scale iButton temperature loggers were at least in part due to the fact that they were exposed to open air and varying degrees of direct sunlight relative to the macro-scale HOBO temperature loggers, which were housed inside a solar shield (i.e., were fully shaded). Micro-scale temperatures under-predicted development time to mature F1 adults by a mean of 4.6 days across all five sites (Fig 3B). Macro-scale estimates followed a similar pattern, with a mean under-prediction of 1.8 days to mature F1 adults across all sites (Fig 3B). However, Mann-Whitney U test results showed no significant differences between observed and predicted degree-day development at micro- or macro-scales at three of the five farms (Fig 3A and Fig 3B). Thus, macro-scale temperature monitoring provides suitable data for degree-day calculations and CBB development predictions.

### Individuals per berry

The total number of individuals per berry was generally lower at high elevation sites throughout the sampling period relative to low elevation sites. Although we did not find a significant correlation between temperature and individual life stage abundance or total individuals per berry, our results seem to align with those of Marino et al. [35] who found significant positive correlations between temperature and the abundance of pupae and adults per berry in Puerto Rico. It is possible that two sampling issues impacted our findings. Firstly, at the end of our sampling period for all locations except the low-elevation Kona site we noted a continuing upward trend in the number of mature adults per berry such that we may not have captured peak mature adult abundance. Secondly, our sampling method did not prevent the potential escape of mature females emerging from infested berries. In order to accurately capture the peak number of adults per berry, future studies should extend the sampling period until the end of the main harvest (December in Hawaii), as well as cover branches with entomological sleeves once the required number of degree days is reached for adults to mature (38–59 days in the present study).

### Development rates and degree-day estimates

CBB development rates in commercial coffee fields varied along an elevational gradient. A complete development cycle (from infestation to the presence of mature F1 adults) was two weeks longer at high-elevation sites (*Tmean* ~20˚C) than at low-elevation sites (*Tmean* ~23˚C). Previous field and laboratory research on CBB development cycles have observed analogous trends along temperature gradients [20, 21, 23, 24, 36], though specific time requirements are variable (Table 4). Overall, previous estimates for development time from egg to adult are ~4–9 days longer than our estimates for sites at 20˚C, ± 3–5 days of our estimate for sites at 22˚C, and ± 1–10 days of our estimate for sites at 23˚C (Table 4).

**Table 4:** Estimated coffee berry borer development times (in days) across a temperature gradient from egg to adult. Times were estimated under field conditions in the present study, as well as in Baker et al. [20] and Ruiz-Cárdenas & Baker [21], while Jaramillo et al. [23] and Giraldo-Jaramillo et al. [24] estimated development times in the laboratory under constant conditions.

Mean Temp (˚C)	Baker et al. 1992	Ruiz-Cárdenas & Baker 2010	Jaramillo et al. 2010	Giraldo-Jaramillo et al. 2018	Present study: teneral adult	Present study: mature adult
20	--	--	53.7	55.9	46.7	49.5
21	49	49	--	--	--	--
22	--	--	--	38.7	35.0	43.4
23	42	--	31.2	--	32.1	36.9

The variability in development rates across these results is likely due to differences in the daily range of temperatures. Faster insect development times have been reported under fluctuating (diurnal) temperatures compared to constant temperatures [37, 38, 39]. However, the range of temperature fluctuations can also influence development at different mean temperatures. Kingsolver et al. [40] reported that at a mean temperature of 20˚C, development time of the tobacco hornworm (*Manduca sexta* L.) decreased with increasing diurnal temperature range, especially at later developmental stages, whereas at a mean temperature of 30˚C, increasing the diurnal temperature range increased development time. In the present study, we observed slower development times at sites that experienced a higher daily temperature range, despite similar mean daily temperatures (Table 3). The Ka‘u and Kona high-elevation sites had similar mean daily temperatures (20.17˚C and 20.67˚C, respectively), but slower development times were observed at the Ka‘u high-elevation site, likely due to a higher range in mean daily temperature (Ka‘u: 14.90˚C vs. Kona: 10.67˚C). This same trend was observed for the two low-elevation sites at a mean daily temperature of ~23˚C (Table 3), as well as for the additional farms we used to calculate CBB generations in Kona and Ka‘u, with Ka‘u farms consistently having slower development rates relative to Kona farms at similar elevations (~300, 500, and 600 m).

### Generations per season

The number of CBB generations per month ranged from 0.33–0.78 for 12 commercial coffee farms on Hawai‘i Island that spanned an elevational gradient of ~600 m. For a typical coffee farm in Kona and for coffee farms in Ka‘u that strip-pick (thus creating a break in the year-round growing season), berries with sufficient dry matter content for CBB reproduction are available from around July–December. This translates to 2.11–3.27 CBB generations per season for high-elevation farms (~600–780 m) and 4.13–4.96 CBB generations per season for low-elevation farms (~200–300 m).

Our estimate of 2.11–3.27 generations per season for high-elevation farms on Hawai‘i Island is within the range predicted by Jaramillo et al. [22] for farms in Colombia, Ethiopia, Kenya, and Tanzania (0.0–4.71 generations/season). For low-elevation farms on Hawai‘i Island, our estimate of 4.13–4.96 generations per season is near the higher end of the range reported by Jaramillo et al. [22], and just outside the lower end of the range reported for São Paulo, Brazil (5.09–10.53 generations/season) by Giraldo-Jaramillo et al. [24]. Given that coffee is a finite resource, Jaramillo et al. [22] posited that regions experiencing temperatures close to the optimum for CBB development (26˚C) throughout the season could experience increased dispersal as more females compete for berries in which to oviposit. Farms at low elevations on Hawai‘i Island that experience a narrow daily range of warm temperatures throughout the coffee-growing season may thus be the subject of more intense pressure from CBB given the faster development rates, high numbers of generations per season, and increased propensity for dispersal.

### A novel technique for inducing CBB infestation in the field

To our knowledge, this is the first experiment using alcohol lures to promote infestation of coffee berries with CBB that are naturally occurring in the field. This technique differs from the conventional “forced infestation” method that releases CBB from lab colonies into entomological sleeve cages surrounding coffee branches [7, 20, 41, 42]. Our technique has some advantages over the conventional method. Firstly, CBB that are naturally occurring in the field are better adapted to the local environment compared to CBB that are reared in lab colonies. Secondly, the use of alcohol lures to attract CBB to coffee berries is more representative of the natural conditions under which infestation takes place in the field, as opposed to forcing infestation by releasing CBB into entomological sleeves. Lastly, entomological sleeves alter the microclimate surrounding coffee berries by promoting high relative humidity, such that there is an increased risk of CBB infection by *B*. *bassiana* during the infestation process (3–8 days). Other studies have reported incidence of *B*. *bassiana* induced mortality in field trials using entomological sleeves for artificial infestation by CBB [7, 43].

A potential limitation in our use of this technique was that we induced infestations over a one-week period, which resulted in a window of 7 days over which berry infestation could have occurred. To account for this issue, we adjusted our development time estimates by 3.5 days (approximately 7% of the average development time to mature adults), and it is possible that this could have affected our comparisons of observed vs. predicted development times. Future studies desiring increased accuracy for development time estimates should check berries daily for infestation.

### Application to IPM programs

Our field validation of laboratory estimates of CBB development rates are an important first step in understanding how CBB populations respond to temperature differences under natural conditions. This information can be incorporated into existing IPM programs as a way to predict when females will initiate a new infestation cycle, and thereby assist with the timing of *B*. *bassiana* sprays early in the coffee season. We suggest a two-phase strategy to integrate the degree day model described here into current CBB management programs. During the first phase, coffee fields are monitored bi-weekly for CBB activity using alcohol-baited traps and/or infestation assessments of berries starting ~30 days after the first flowering of the season [14, 15, 31]. If CBB is present at or above the recommended economic threshold (e.g., the Cenicafé threshold is 5% infestation), management is conducted by using sprays of *B*. *bassiana* or other pesticides approved for coffee [13, 14, 15, 44]. When the first major peak in CBB flight activity of the growing season is detected, the second phase is initiated, and degree-day accumulations begin. Once the total degree day accumulation reaches 332-degree days, pesticide sprays can be applied such that they coincide with emergence of the second generation. Degree-day accumulation would then be re-started to predict emergence of the third generation and so on. This strategy has the advantage of controlling CBB populations early in the growing season, which has been shown to be critical for minimizing crop damage for the remainder of the season [19]. This two-phase method will also help reduce pesticide inputs into the environment, as well as lower labor and production costs for the grower as pesticides will only need to be applied at certain points in the year rather than using regularly scheduled calendar sprays [17, 19]. A potential caveat to this may exist in that some coffee-growing regions experience year-round flowering and fruiting (e.g., Ka‘u on Hawai‘i Island), and this lack of a defined start to the coffee season may lead to difficulties in setting the start date for CBB development times using degree-days and the subsequent timing of pesticide sprays [14]. Applying foliar sprays of plant growth regulators such as gibberellic acid could help to synchronize flowering and coffee berry development in such regions [45]. An effective strategy for managing CBB will involve monitoring coffee flowering phenology along with CBB flight activity, followed by using degree-day calculations to estimate female emergence and determine appropriate timing for pesticide applications [46].

## Conclusions

The principal aim of our study was to aid in improving coffee berry borer IPM by validating and refining laboratory degree-day predictions for CBB development rate by collecting real-time data on micro- and macro-scale weather while simultaneously tracking CBB development across an elevational gradient. Using macro-scale temperature data from five sites on Hawai‘i Island and laboratory degree-day models, our study established a mean requirement of 332 (LTT = 14.9°C) and 386 (LTT = 13.9°C) degree-days from the time of berry infestation to the maturation of female F1 offspring. Field validation of the LTT for CBB reproduction and pre-reproductive degree-day models developed by Jaramillo et al. [22] and Giraldo-Jaramillo et al. [24] suggest that these laboratory estimates are applicable to dynamic field settings and are useful for predicting CBB infestation cycles under varying field conditions. Secondarily, we found a predictive relationship between elevation and the number of generations per month for CBB in Hawaii, which can be used to help predict population dynamics at coffee-growing sites in the future.
